# Implementation of a Quantum Authentication Protocol Using Single Photons in Deployed Fiber

**DOI:** 10.3390/e28040366

**Published:** 2026-03-24

**Authors:** Changho Hong, Youn-Chang Jeong, Se-Wan Ji

**Affiliations:** The Affiliated Institute of ETRI, Yuseong-daero 1559, Yuseong-gu, Daejeon 34044, Republic of Korea; w3140@nsr.re.kr (Y.-C.J.); sewanji@nsr.re.kr (S.-W.J.)

**Keywords:** quantum communication, quantum network, quantum authentication

## Abstract

With the increasing importance of securing quantum communication networks, practical and robust entity authentication is a critical requirement. Accordingly, we propose and experimentally validate a quantum entity authentication (QEA) protocol specifically designed for integration with BB84-type quantum key distribution (QKD) workflows and existing terminal architectures. We analyze the protocol’s security against intercept–resend man-in-the-middle (MitM) impersonation, showing that an unauthenticated adversary induces a characteristic 25% correlation error and that the rejection probability approaches unity as the number of detected authentication events increases. For practical realization, the protocol is deployed using weak coherent pulses (WCPs) with decoy-state estimation to bound single-photon contributions and mitigate photon-number-splitting (PNS)-enabled leakage. The system is demonstrated over a field-deployed fiber link of approximately 20 km with ~8 dB optical loss using signal/decoy intensities of ~0.5/~0.15 and sending probabilities 0.88/0.10/0.02 (signal/decoy/vacuum). Across both verification directions, stable operation is observed with quantum bit error rate (QBER) typically fluctuating between 1% and 4% while the sifted key rate remains constant over time. These results provide an experimental basis for integrating physical-layer entity authentication into deployed quantum communication networks.

## 1. Introduction

The proliferation of quantum information infrastructures has catalyzed the development of a global quantum internet, promising a paradigm shift in secure communication through the principles of quantum mechanics [1,2]. While QKD has reached a certain level of maturity, the overarching security of any quantum-cryptographic framework is fundamentally predicated on the robustness of entity authentication [3,4]. Without a rigorous mechanism to verify the legitimacy of communicating parties, even channels with information-theoretic security remain susceptible to MitM and impersonation attacks [5,6]. As the advent of fault-tolerant quantum computers threatens the computational hardness underlying classical public-key infrastructures (PKI), there is increasing interest in authentication protocols whose security is derived from the laws of physics [7,8].

In 2020, the U.S. National Security Agency (NSA) explicitly stated that QKD itself provides no mechanism to authenticate the source of a QKD transmission and that entity authentication therefore requires either asymmetric cryptography or preplaced keys [9]. This position clarifies that the practical vulnerabilities of QKD deployments may hinge on whether peer identity is assured, and it further implies that the overall security level of QKD-based communication systems is determined by the assumptions underpinning the authentication layer. Traditionally, QKD systems have relied on information-theoretic message authentication codes (MACs) based on universal hashing—most notably the Wegman–Carter family—seeded with a short pre-shared symmetric key [10]. The Wegman–Carter paradigm offers an “unconditional” authentication notion that statistically bounds forgery even against an adversary with unbounded computational power. Moreover, researchers have systematically examined the conditions and limitations for economizing and recycling authentication keys, supporting feasibility analyses in the QKD setting [11]. From the perspective of large-scale networking and scalability, however, pre-shared-key (PSK) authentication entails bootstrap requirements as well as key distribution and management overhead. As a practical alternative, several studies have proposed and experimentally explored integrating PKI and post-quantum cryptography (PQC) into the authentication layer of QKD deployments [12]. PQC standardization has been pursued on the basis of mathematical problems believed to remain hard in the presence of quantum computing (e.g., structured lattices and hash-based constructions) [13]. Hybridizing computational authentication with QKD facilitates near-term deployment while leveraging long-term, QKD-derived secrecy [12,14]. However, unlike QKD’s information-theoretic framework, this approach relies on computational assumptions for identity assurance, creating a heterogeneous security profile. Given the quantum threat to classical cryptography, placing the entire trust chain on computational premises may conflict with long-term goals of enduring confidentiality and sustained trust [7].

Against this backdrop, there is strong motivation to align the entity authentication required for QKD operation with its underlying security objectives, including strong adversarial models and physics-based guarantees. When the authentication layer relies on assumptions that diverge from those underlying QKD’s security claim, the resulting end-to-end security argument can become fragmented [9,15,16]. In this sense, the proposed QEA protocol strengthens end-to-end security in QKD-based communications by addressing, from a physics-based standpoint, the foundational question of “with whom the secret key is securely shared.”

The theoretical landscape of quantum-based authentication was pioneered by Barnum et al., who established the foundational criteria for the non-malleability of quantum states [17]. While early research focused extensively on quantum message authentication (QMA), the conceptual framework has evolved toward QEA and identification [18,19]. In these schemes, the no-cloning theorem provides a physical guarantee that an adversary cannot replicate the “quantum credentials” or “unclonable tokens” assigned to a legitimate user [20,21]. Various identification protocols using entangled states or single-photon measurements have been proposed to ensure that identity verification does not leak information that could be exploited in subsequent sessions [22,23]. Recently, these frameworks have been further expanded to complex network environments, such as protocols for simultaneous multiparty authentication involving classical third parties [24].

Despite these theoretical advancements, a gap remains between idealized protocols and their physical realization in practical communication channels. A significant portion of existing experimental literature relies on WCP as a surrogate for single photons [25,26]. However, the Poissonian photon-number distribution of WCPs introduces a critical vulnerability: PNS attack, where an eavesdropper can intercept redundant photons to gain partial information about the user’s identity without being detected [27,28]. Furthermore, environmental decoherence and channel attenuation in optical fibers or free-space links degrade the signal-to-noise ratio (SNR), often leading to an increase in false rejection rates (FRR) in real-world deployments [29,30].

This paper contributes to advancing the practical realization of quantum networks by implementing and experimentally validating a quantum authentication protocol in a deployed communication setting. Our protocol adopts a challenge–response architecture in which the user’s identity is encoded in polarization degrees of freedom and is designed to be executable within the operational framework of BB84-type QKD without requiring changes to the overall system architecture. We provide an analysis of the authentication success rate and associated security bounds under realistic channel noise and detector inefficiencies. These results support the feasibility of integrating physical-layer authentication into next-generation quantum networks, so that user legitimacy can be verified under the same physics-based threat considerations that motivate quantum-secure communication [4,30].

From both security and engineering standpoints, the proposed protocol offers several practical advantages. First, the proposed scheme is a lightweight, PSK-seeded challenge–response procedure that can be executed within a BB84-type QKD operation either periodically or on an event-driven basis. One-way entity authentication is obtained by running a single verification direction, while mutual authentication can be realized as an optional extension by executing the protocol in both directions when required, without modifying the existing optical hardware or disrupting the QKD workflow. Second, because the shared PSK implicitly determines the measurement basis (MB), a MitM adversary is forced to guess, inducing characteristic correlation errors. This allows the verifier to drive the rejection probability near unity by accumulating authentication events, even under intrinsic channel QBER. Third, the protocol is deployment-ready: while defined for single photons, it supports practical weak coherent pulse (WCP) transmitters via decoy-state estimation. This suppresses photon-number-splitting (PNS) attacks, retaining idealized security guarantees over realistic channels [25,26,27,28,31,32,33,34]. Finally, although demonstrated with polarization encoding, the protocol naturally maps to phase-based encodings, enabling seamless integration as a physical-layer authentication module in standard QKD infrastructures [35].

While a 25% disturbance signature under basis guessing is well known from the BB84 intercept–resend intuition, its role in the present work is different. Here, the disturbance is exploited as a physical-layer test of PSK possession and therefore entity authenticity, rather than as evidence of secrecy of a newly generated key. In our protocol, the MB is deterministically selected from the shared PSK (instead of being randomly chosen and later reconciled), which enables a lightweight challenge–response authentication procedure that reuses BB84 terminal control logic without architectural modification. We further connect experimentally observable quantities (gain and QBER in a WCP/decoy implementation) to an explicit acceptance rule and finite-size authentication bounds, thereby providing a deployable and quantitatively analyzable authentication primitive for QKD-based networks.

The advantage of the proposed protocol under the present trust model should not be interpreted as generating a larger amount of secret bits from the initially provisioned PSK than would be possible by one-time injection of a much longer static key. Rather, the proposed protocol is intended as a deployable entity-authentication mechanism whose operational significance is that authentication keys can be updated during runtime within the BB84-type QKD workflow. This distinction is important because the relevant quantity in our analysis is the authentication security strength against impersonation, not the volume of newly generated secret key material. From a key-management viewpoint, such runtime updating is meaningful because practical systems generally restrict key lifetime and incorporate fresh keying material over time, instead of relying indefinitely on a single long-lived static authentication key [36,37,38].

The main contributions of this work are:

- We propose a PSK-seeded challenge–response QEA protocol that is directly compatible with BB84-type QKD terminal architectures and workflows.
- We analyze acceptance and rejection behavior under a QBER-threshold decision rule and derive explicit bounds for FRR and false acceptance rate (FAR) in a finite-size setting.
- We extend the analysis to a practical WCP realization by incorporating standard decoy-state estimation to conservatively quantify the usable single-photon authentication trials and to mitigate PNS-enabled leakage.
- We experimentally validate the protocol over a ~20 km field-deployed fiber link with ~8 dB loss and report stable operation with QBER typically in the 1–4% range.
- We discuss PSK consumption and present an information-theoretic PSK refresh policy using QKD-generated keys, enabling sustainable long-term operation.

The remainder of this paper is organized as follows. Section 2 presents the protocol description and operational steps of the proposed QEA method. Section 3 provides a security evaluation of the proposed protocol, including the analysis framework needed for practical implementations (e.g., bounds in the WCP/decoy-state setting). Section 4 reports on the experimental realization and observed performance metrics over a deployed fiber link. Finally, Section 5 concludes the paper and discusses implications for integrating physical-layer authentication into future quantum communication infrastructures.

## 2. Protocol Description

Entity authentication requires several preconditions. The proposed QEA protocol is described under the following assumptions.

Pre-shared authentication key
Alice and Bob share a pre-distributed entity-authentication key, denoted by $A_{k}$, which is used as a PSK.
Authenticated classical channel
We assume integrity protection for classical messages exchanged during the protocol (nonce announcements/requests and related transcripts). This can be realized using information-theoretic MACs seeded with PSK or QKD-refreshed keys, consistent with the information-theoretic end-to-end security narrative.
State–bit mapping
Each symbol of the PSK is a two-bit string. For the *i*-th symbol ${\left(A_{k}\right)}^{i}\in \;\left\{00,\;01,\;10,\;11\right\}$, the corresponding single-photon quantum state is chosen according to the BB84 state set

(1) $$\left\{\left|\left.0\right\rangle \right.,\left|\left.1\right\rangle \right.,\left|\left.+\right\rangle \right.,\left|\left.-\right\rangle \right.\right\}$$

The mapping is defined as

(2) $$\left|\left.0\right\rangle \right.⇄00,\;\left|\left.1\right\rangle \right.⇄01,\;\left|\left.+\right\rangle \right.⇄10,\;\left|\left.-\right\rangle \right.⇄11.$$

Under this assumption, the purpose of the initial trusted provisioning is not to claim an advantage in raw secret-bit volume over arbitrary one-time installation of a larger static authentication key. Instead, the purpose is to establish an initial authenticated operating point after which the PSK can be refreshed using newly generated QKD secret bits during runtime. The resulting advantage is therefore operational: fresh authentication keys can be applied without continued dependence on one long-lived static authentication key, and the authentication process remains integrated with the active QKD workflow. This is consistent with standard key-management rationale, in which rekeying is used to limit cryptoperiod and to reduce the impact of compromise of old keying material [36,37,38].

Each entity is assigned a unique authentication key, typically issued by a trusted authority. The entity identifier (ID) itself does not represent the PSK; rather, the PSK is a shared secret credential bound to that ID. The protocol is designed to execute either periodically or aperiodically, running concurrently with a BB84-type QKD protocol. We assume that a pre-agreed time or event triggers the authentication procedure; accordingly, this work focuses on the protocol’s operational steps.

We assume that the initial PSK $A_{k}$ is provisioned to Alice and Bob by a trusted authority (TA) using an information-theoretically secure mechanism prior to network operation (e.g., secure offline installation or a physically protected channel). During runtime, the PSK is treated as a consumable secret and is refreshed using fresh, information-theoretically secure bits derived from the secret key generated by the immediately preceding BB84-type QKD session. Concretely, letting $K_{QKD}^{s}$ denote the final secret key of QKD session s (after error correction and privacy amplification), the PSK for the next authentication execution is set by extracting the required number of uniformly random bits from $K_{QKD}^{s}$ and binding them to a public session identifier and an authentication direction label (domain separation). This aligns the authentication bootstrap with the same information-theoretic security narrative as QKD, up to standard device and modeling assumptions.

Let ${\left(A_{k}\right)}^{i}=\left(b_{i},\;v_{i}\right)$ denote the two bits of the i-th PSK symbol, where $b_{i}\in \;\left\{0,1\right\}$ is the first bit and $v_{i}\in \;\left\{0,1\right\}$ is the second bit.

Step 1. State preparation from PSK

Alice generates a single photon and prepares its quantum state according to the shared PSK:

(3) $$\left|\left.\psi_{i}\right\rangle \right.=S\left({\left(A_{k}\right)}^{i}\right)$$

where S(·) is the mapping defined Equation (2). For example, if ${\left(A_{k}\right)}^{i}=10$, then $\left|\left.\psi_{i}\right\rangle \right.=\left|\left.+\right\rangle \right.$.

Step 2. Random nonce operation

Alice samples a random nonce bit $n_{i}\in \;\left\{0,1\right\}$ and applies

(4) $$\left|\left.\phi_{i}\right\rangle \right.=N_{i}\left|\left.\psi_{i}\right\rangle \right.,\;N_{i}\in \left\{I,i\sigma_{y}\right\}$$

We interpret $n_{i}=0$ as selecting $N_{i}=I$ and $n_{i}=1$ as selecting $N_{i}=i\sigma_{y}$.

Step 3. Quantum transmission

Alice transmits the resulting single-photon state $\left|\left.\phi_{i}\right\rangle \right.$ to Bob over the quantum channel.

Step 4. Basis choice and measurement

Bob chooses a MB according to the first bit $b_{i}$ of ${\left(A_{k}\right)}^{i}$:

- If $b_{i}=0$, Bob measures in the z-basis $\left\{\left|\left.0\right\rangle \right.,\left|\left.1\right\rangle \right.\right\}$.
- If $b_{i}=1$, Bob measures in the x-basis $\left\{\left|\left.+\right\rangle \right.,\left|\left.-\right\rangle \right.\right\}$.

Bob stores his measurement outcome for each successfully detected photon.

If the channel were lossless and the nonce operation were not applied, Bob could directly confirm that the PSK $A_{k}$ are deterministically recovered from his measurement outcomes. In practice, channel loss and the deliberate random nonce operation invalidate these simplifying assumptions.

The remaining steps depend on which party plays the verifier and which plays the prover.

Case a: Alice is the verifier and Bob is the prover
Step 5a. Bob reconstructs nonce bits

Using the stored measurement outcomes and $A_{k}$, Bob reconstructs the nonce bit values and announces them to Alice over the public (classical) channel.

A convenient way to express this reconstruction is as follows. Let $m_{i}\in \;\left\{0,1\right\}$ denote Bob’s measurement result encoded as

- $m_{i}=0$ for outcomes $\left|\left.0\right\rangle \right.$ (in z-basis) or $\left|\left.+\right\rangle \right.$ (in x-basis).
- $m_{i}=1$ for outcomes $\left|\left.1\right\rangle \right.$ (in z-basis) or $\left|\left.-\right\rangle \right.$ (in x-basis).

From the action of $N_{i}$, in the ideal setting we have

(5) $$m_{i}=v_{i}⨁n_{i}$$

and Bob can compute

(6) $$\hat{n_{i}}=m_{i}⨁v_{i}$$

Step 6a. Alice verifies Bob

Alice compares Bob’s announced nonce-bit sequence $\left\{\hat{n_{i}}\right\}$ with her locally chosen nonce bits $\left\{n_{i}\right\}$. If the correlation agrees within the intrinsic QBER of the Alice–Bob quantum channel, Alice accepts Bob as authenticated; otherwise, she rejects.

Case b: Bob is the verifier and Alice is the prover
Step 5b. Bob requests nonce disclosure

Bob requests Alice to disclose the nonce bit values corresponding to the nonce operator $\left\{N_{i}\right\}$ applied in Step 2.

Step 6b. Alice discloses nonce bits

Alice discloses the nonce-bit values over the public (classical) channel.

Step 7b. Bob verifies Alice

Bob checks the correlation between the disclosed nonce bits $\left\{n_{i}\right\}$ and his measurement outcomes $\left\{m_{i}\right\}$. If the correlation agrees within the intrinsic QBER of the Alice–Bob quantum channel, Bob accepts Alice as authenticated; otherwise, he rejects.

**Figure 1:**
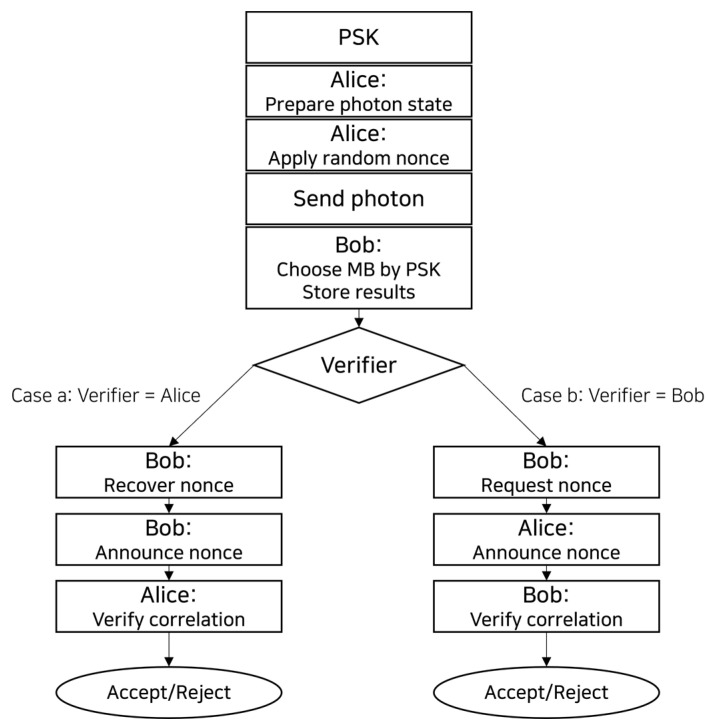
Flowchart of the proposed QEA protocol. The protocol supports two verification directions; mutual authentication can be obtained, when desired, by executing both directions, while one-way authentication uses only a single direction depending on the verifier role.

Section 3.3.1 offers a more in-depth analysis of the acceptance rule. The overall procedure of the protocol is illustrated in Figure 1.

For implementation validation on a deployed fiber, the proposed protocol adopts WCPs and the decoy-state technique commonly used in QKD. Further details are discussed in a subsequent Section 3.2.

## 3. Security Evaluation of the Proposed Protocol

We consider an adversary who has full control over the quantum channel (blocking, injecting, and performing arbitrary measurements, including with quantum memory), while the classical channel is assumed to be authenticated but not encrypted. In our setting, the “authenticated classical channel” assumption can be instantiated by information-theoretic message authentication (e.g., universal-hash/Wegman–Carter MACs) seeded with pre-shared or QKD-refreshed secret bits; the corresponding secret-key consumption is explicitly accounted for in the key-budget term $R_{MAC}$ in Section 3.3.4. The adversary does not know the PSK. Under this model, the primary goals of Eve are (i) impersonation of a legitimate entity (false acceptance) and/or (ii) learning PSK material over repeated protocol executions. We explicitly separate (a) MitM relay/intercept–resend attacks that disturb an otherwise honest exchange, from (b) pure impersonation attacks in which Eve acts as the prover without possessing the PSK. We additionally discuss how practical WCP emission statistics and nonce disclosure interact with long-term PSK management.

### 3.1. Security Analysis of MitM Attacks

The security of the proposed protocol relies fundamentally on the secrecy of the PSK, denoted as $A_{k}$, which is distributed beforehand between Alice and Bob. In this analysis, we assume the public classical channel is authenticated but not encrypted, meaning an adversary can observe but not modify classical messages without detection. Consequently, the primary attack surface available to an external adversary (Eve) is the quantum channel.

Eve’s primary objective is to impersonate either Alice or Bob (impersonation attack) or to extract information regarding $A_{k}$. To achieve this, Eve typically employs a MitM strategy, specifically the intercept–resend attack, where she intercepts the quantum signals transmitted over the channel, measures them, and resends new states to the recipient. Since the protocol can be executed in either verification direction (Alice verifies Bob, or Bob verifies Alice), we evaluate security for both directions. Mutual authentication is an optional use case obtained by running both directions, whereas one-way authentication requires only a single direction.

Consider the scenario where Alice acts as the verifier and Bob as the prover. Eve intercepts the quantum signal transmitted by Alice and performs a measurement to forge a valid response. However, since Eve does not possess the PSK ($A_{k}$), she has no knowledge of the correct MB for each signal. Consequently, she must choose a basis at random. When Eve measures in the wrong basis (which happens with a probability of 1/2) and resends the resulting state, Bob—who measures in the correct basis determined by $A_{k}$—will obtain a random result. This process introduces an error rate of 25% in the reconstructed nonce bits compared to the original bits sent by Alice. In Step 6a, Alice compares the nonce sequence reconstructed by the prover with her local values. The probability that Eve successfully passes this verification without triggering an error is ${\left(\frac{3}{4}\right)}^{D}$, where D is the number of successfully detected quantum states used for authentication. Therefore, the probability that the protocol rejects an unauthenticated adversary is given by:

(7) $${\left(P_{f}\right)}^{Bob}=1-{\left(1-\frac{1}{4}\right)}^{D}$$

As D increases, the rejection probability ${\left(P_{f}\right)}^{Bob}$ converges to 1, ensuring unconditional security against the intercept–resend attack.

In the reverse scenario where Bob verifies Alice, the security logic remains symmetric. If Eve attempts to impersonate Alice by sending forged quantum states, she must again guess the basis defined by $A_{k}$. Bob, measuring these states according to the correct PSK, will observe a 25% error rate in the correlation between his measurement outcomes and the nonce values subsequently disclosed by Eve (or forged by Eve). Accordingly, the probability of Bob rejecting an illegitimate prover in Step 7b is:

(8) $${\left(P_{f}\right)}^{Alice}=1-{\left(1-\frac{1}{4}\right)}^{D}$$

In an ideal lossless environment, the number of authentication bits D would equal half the length of the used PSK string (i.e., $D∼\;\left|A_{k}\right|/2$). However, in practical implementations, channel loss and detector inefficiency significantly reduce the number of valid detection events. The parameter D is analogous to the concepts of yield and overall gain used in QKD performance analysis. The yield is defined as the conditional probability that a signal sent by Alice results in a detection by Bob. The overall gain ($Q_{\mu }$) represents the ratio of the total number of detected signals to the total number of transmitted pulses. Consequently, the effective length D corresponds to the number of final detected events at Bob’s side. In typical QKD systems operating over standard optical fiber distances, the overall gain $Q_{\mu }$ is often observed in the order of $10^{-3}$ or lower depending on the transmission distance [31,32,33]. Therefore, to achieve a sufficiently high security parameter D (and thus a rejection probability close to 1), the protocol must transmit a sufficient number of pulses to compensate for the system’s overall attenuation.

The analysis in this paper focuses on authentication security stemming from PSK-seeded basis control under the above channel model, and on implementation security captured by standard decoy-state bounds in the WCP setting. Full treatment of device-level side-channel attacks (e.g., detector blinding, Trojan-horse probing of modulators, and calibration attacks) is out of scope here; mitigating such attacks requires device-countermeasure techniques orthogonal to the present protocol logic. We include this statement to clearly delimit the security guarantees claimed in this work.

### 3.2. Security Analysis Within a QKD Realization Framework

Although the protocol is defined in the single-photon setting, its communication-channel implementation adopts WCPs and the decoy-state method. We follow the standard decoy-state modeling adopted by Park et al. [34] to lower-bound the single-photon contribution, since multi-photon events can enable PNS-type leakage and are therefore treated as not contributing to secure authentication strength. Section 3.2.1, Section 3.2.2 and Section 3.2.3 briefly recall standard WCP channel/detector modeling and decoy-state estimation that are widely used to bound single-photon gain and error terms. The protocol-specific step in this work starts from Section 3.2.4, where we translate these standard bounds into a finite-size lower bound on the number of usable authentication trials for our acceptance test; this quantity then parameterizes the FRR/FAR exponents and the security strength κ in Section 3.3.

#### 3.2.1. Channel/Detector Model and Overall Gain

Let $t_{AB}$ be the channel transmittance, $\eta_{Bob}$ represent Bob-side component transmission, $\eta_{D}$ be detector efficiency. Define the overall transmission efficiency

(9) $$r=t_{AB}\eta_{Bob}\eta_{D}$$

When fiber loss is α dB/km over distance l km and Bob-side component loss is β dB,

(10) $$t_{AB}=10^{-\alpha l/10},\;\eta_{Bob}=10^{-\beta /10}.$$

Let $Y_{0}$ be the background (vacuum) yield (dark counts + background clicks). For an i-th photon state, we use the yield model

(11) $$Y_{i}=1-\left(1-Y_{0}\right){\left(1-r\right)}^{i}.$$

For a WCP with mean photon number x (e.g., signal x=μ, decoy x=ν), the photon-number distribution is Poisson:

(12) $$P_{x}\left(i\right)=e^{-x}\frac{x^{i}}{i!}.$$

The overall gain (click probability) is

(13) $$Q_{x}=\sum_{i=0}^{\infty }P_{x}\left(i\right)\;Y_{i}=\sum_{i=0}^{\infty }e^{-x}\frac{x^{i}}{i!}\left[1-(1-Y_{0}){\left(1-r\right)}^{i}\right].$$

Substituting the yield model $Y_{i}=1-\left(1-Y_{0}\right){\left(1-r\right)}^{i}$ into the Poisson mixture $Q_{x}=\sum_{i}P_{x}\left(i\right)Y_{i}$, we separate the sum into the trivial normalization term and a residual term weighted by ${\left(1-r\right)}^{i}$. The latter is the exponential generating function of the Poisson distribution, which can be evaluated in closed form. For simplification of $Q_{x}$, we split the sum:

(14) $${\;\;\;\;Q}_{x}=\sum_{i=0}^{\infty }e^{-x}\frac{x^{i}}{i!}-\left(1-Y_{0}\right)\sum_{i=0}^{\infty }e^{-x}\frac{x^{i}}{i!}\;{\left(1-r\right)}^{i}\;\;\;\;=1-\left(1-Y_{0}\right)e^{-x}\sum_{i=0}^{\infty }\frac{{\left(x(1-r)\right)}^{i}}{i!}.$$

Use $\sum_{i=0}^{\infty }\frac{\alpha^{i}}{i!}=\;e^{a}$:

(15) $$e^{-x}\sum_{i=0}^{\infty }\frac{{\left(x(1-r)\right)}^{i}}{i!}=e^{-x}e^{x(1-r)}=e^{-xr}.$$

Hence,

(16) $$Q_{x}=1-\left(1-Y_{0}\right)e^{-xr}.$$

#### 3.2.2. QBER Model

Let $e_{0}=1/2$ be the error probability of background clicks (random outcomes), and let $e_{d}$ be the intrinsic misalignment/detection error probability for true signal clicks. Then

(17) $$E_{x}Q_{x}=e_{o}Y_{o}+e_{d}\left(Q_{x}-Y_{o}\right).$$

Substituting (16) gives

(18) $$E_{x}Q_{x}=e_{o}Y_{o}+e_{d}\left(1-\left(1-Y_{0}\right)e^{-xr}-Y_{0}\right)\;=e_{o}Y_{o}+e_{d}\left(1-Y_{0}\right)\left(1-e^{-xr}\right).$$

#### 3.2.3. Decoy-State Bounds for Single-Photon Terms

Use three intensities: signal μ, weak decoy ν, and vacuum 0 (0<ν<μ). Let the observed gains and QBERs be $Q_{\mu }$, $Q_{\nu }$, $Q_{0}$, $E_{\mu }$ and $E_{\nu }$. From vacuum decoy, $Y_{0}=Q_{0}$. Multiply by $e^{x}$ to obtain the standard decoy expansion:

(19) $$Q_{x}e^{x}=\sum_{i=0}^{\infty }Y_{i}\frac{x^{i}}{i!}.$$

In particular,

(20) $$Q_{\mu }e^{\mu }=Y_{o}+Y_{1}\mu +Y_{2}\frac{\mu^{2}}{2!}+Y_{3}\frac{\mu^{3}}{3!}+\cdots ,\;\;Q_{\nu }e^{\nu }=Y_{o}+Y_{1}\nu +Y_{2}\frac{\nu^{2}}{2!}+Y_{3}\frac{\nu^{3}}{3!}+\cdots .$$

To obtain a conservative lower bound on $Y_{1}$, we apply the standard three-intensity (vacuum + weak decoy) decoy-state elimination: we form a linear combination of the expansions for μ and ν to cancel the higher-photon contributions. Since $Y_{i}\geq 0$ for i≥2, discarding the remaining multi-photon terms yields the lower bound $Y_{1}\geq Y_{1}^{L}$ in Equation (21).

A conservative lower bound on the single-photon yield $Y_{1}$ (vacuum + weak decoy) is

(21) $$Y_{1}\geq Y_{1}^{L}=\frac{\mu }{\mu \nu -\nu^{2}}\left(Q_{\nu }e^{\nu }-\frac{\nu^{2}}{\mu^{2}}Q_{\mu }e^{\mu }-\frac{\mu^{2}-\nu^{2}}{\mu^{2}}Y_{0}\right).$$

Then the single-photon gain satisfies

(22) $$Q_{1}=Y_{1}\mu e^{-\mu }\;\;\;\;\;⟹\;\;\;\;\;Q_{1}\geq Q_{1}^{L}=Y_{1}^{L}\mu e^{-\mu }.$$

Similarly, using the error gain expansion

(23) $$E_{x}Q_{x}e^{x}=\sum_{i=0}^{\infty }e_{i}Y_{i}\frac{x^{i}}{i!}.$$

An upper bound on the single-photon error rate follows analogously by applying the same decoy-state bounding logic to the error-gain expansion $E_{x}Q_{x}e^{x}$, and then dividing by the lower-bounded single-photon contribution. An upper bound on the single-photon error rate is

(24) $$e_{1}\leq e_{1}^{U}=\frac{E_{\nu }Q_{\nu }e^{\nu }-e_{0}Y_{0}}{Y_{1}^{L}\nu }.$$

#### 3.2.4. Lower Bound on Usable Authentication Trials

In the finite-size estimation below, we use the standard i.i.d. (independent and identically distributed) trial model that is widely adopted in decoy-state QKD analyses. Concretely, after conditioning on the chosen intensity class, each pulse is regarded as an identical repetition of the same channel–detector experiment (same parameters and hence the same single-trial distribution), and different pulses are assumed to produce independent outcomes. This stationarity-and-independence idealization allows the number of usable single-photon authentication events to be modeled with binomial statistics and supports the normal/Chernoff-type approximations applied in this subsection. When non-stationary behavior or memory effects are present, a more general correlated-trial finite-size analysis would be needed, which is beyond the scope of this work.

Let N be the number of WCP pulsed used for one execution of the authentication procedure (i.e., the number of indices i for which Alice prepares $\left|\left.\phi_{i}\right\rangle \right.$ and Bob attempts detection/measurement). Define the random variable X as the number of detected single-photon authentication events among these N pulses. Under independent trials, $X\;∼\;Binomial\left(N,\;Q_{1}\right)$. Since $Q_{1}\geq Q_{1}^{L}$, a conservative approximation uses $Q_{1}^{L}$. For sufficiently large N, apply a normal approximation:

(25) $$X\approx N\left(NQ_{1}^{L},NQ_{1}^{L}\left(1-Q_{1}^{L}\right)\right).$$

Let $z_{a}$ be the standard normal quantile for a one-sided confidence level 1−α (0<α<1). Then a finite-size lower bound on usable trials is

(26) $$D=NQ_{1}^{L}-z_{a}\sqrt{NQ_{1}^{L}\left(1-Q_{1}^{L}\right)}.$$

It means that with confidence at least 1−α at least D detected single-photon events are available for the authentication decision rule in Section 3.3.

### 3.3. Security Analysis for the Authentication Protocol Under QKD Framework

#### 3.3.1. Acceptance Rule (QBER-Threshold)

Let I be the set of indices used for verification, with $\left|I\right|=D$ as lower-bounded in (26). Define the mismatch indicator

(27) $$\Delta_{i}=\left\{\begin{array}{c} 1\left\{\hat{n_{i}}\neq n_{i}\right\}\;\;\;\;\;\;\;\;\;\;\;\;\;\;\left(Case\;a:Alice\;virifies\;Bob\right),\\ 1\left\{m_{i}\neq v_{i}⨁n_{i}\right\}\;\;\;\;\;\left(Case\;a:Bob\;virifies\;Alice\right), \end{array}\right.$$

where $m_{i}\in \left\{0,1\right\}$ is Bob’s measurement bit as defined in Section 2, $v_{i}$ is the value bit of the PSK symbol $\left(b_{i},\;v_{i}\right)$, and $n_{i}$ is the nonce bit. The total mismatch count is

(28) $$W=\sum_{i\subset I}\Delta_{i}.$$

The verifier accepts if the mismatch rate is below a threshold $\tau \in \left(0,\;\frac{1}{2}\right)$:

(29) $$\frac{W}{D}\leq \tau ,(equivalently\;W\leq \tau D).$$

#### 3.3.2. Completeness: False Rejection Probability

To upper-bound the false-rejection probability in finite size, we assume an i.i.d. mismatch process on the verified index set. Under this assumption, each verified position corresponds to an independent repetition of the same mismatch experiment with a constant per-trial mismatch probability (i.e., the trials share the same distribution), so that the total mismatch count admits a binomial model and standard Chernoff/normal tail bounds become applicable. This is the conventional simplifying assumption in decoy-state-based performance/security estimates when operating conditions are approximately stationary over the analyzed block. A rigorous treatment that allows general temporal correlations or memory would require stronger finite-key tools and is left for future work.

Assume an honest prover and verifier. Let the per-trial mismatch probability on the verified set be $e_{auth}$, dominated by the single-photon error rate. Conservatively,

(30) $$e_{auth}\leq e_{1}^{U},$$

where $e_{1}^{U}$ is estimated in (24). Under independent trials,

(31) $$W∼Binomial\left(D,e_{auth}\right).$$

The FRR is therefore

(32) $$P_{FRR}=Pr\left[W>\tau D\right]\leq \sum_{k=\left\lfloor \tau D\right\rfloor +1}^{D}\left(\begin{array}{c} D\\ k \end{array}\right){\left(e_{auth}\right)}^{k}{\left(1-e_{auth}\right)}^{D-k}.$$

The binomial tail in Equation (32) admits a standard Chernoff-type bound; expressing the exponent via the binary relative entropy $KL\left(\tau ∥e_{auth}\right)$ makes the D-scaling explicit. For large D, we additionally report the normal approximation in Equations (35) and (36) as an intuitive accuracy/engineering estimate.

For $\tau >e_{auth}$, a Chernoff bound yields

(33) $$Pr\left[W>\tau D\right]\leq exp\left(-D\cdot KL\left(\tau ∥e_{auth}\right)\right),$$

where the binary relative entropy is

(34) $$KL\left(\tau ||e\right)=\tau ln\left(\frac{\tau }{e}\right)+\left(1-\tau \right)ln\left(\frac{1-\tau }{1-e}\right).$$

For large D,

(35) $$W\approx N\left(De_{auth},De_{auth}(1-e_{auth})\right),$$

so

(36) $$P_{FRR}\approx 1-\Phi \left(\frac{\tau D-De_{auth}}{\sqrt{De_{auth}\left(1-e_{auth}\right)}}\right)=1-\Phi \left(\frac{\tau -e_{auth}}{\sqrt{e_{auth}\left(1-e_{auth}\right)/D}}\right),$$

where Φ(·) is the standard normal cumulative distribution function (CDF).

#### 3.3.3. Soundness: Impersonation Success Probability

We analyze impersonation under the standard assumption that the PSK bits ${\left\{\left(b_{i},\;v_{i}\right)\right\}}_{i\subset I}$ are unknown to the adversary and are computationally indistinguishable from uniform (or information-theoretically uniform if provisioned as such). In particular, the value-bit is unbiased:

(37) $$Pr\left[v_{i}=0\right]=Pr\left[v_{i}=1\right]=\frac{1}{2}.$$

In the ideal relation $m_{i}=\nu_{i}⨁n_{i}$, for any fixed $m_{i}\in \left\{0,1\right\}$ we have

(38) $$Pr\left[n_{i}=0|m_{i}\right]=Pr\left[v_{i}=m_{i}|m_{i}\right]=\frac{1}{2},\;\;Pr\left[n_{i}=1|m_{i}\right]=Pr\left[v_{i}\neq m_{i}|m_{i}\right]=\frac{1}{2}.$$

Hence, without knowledge of $v_{i}$, the adversary’s optimal single-bit guessing probability for the correct nonce value is bounded by

(39) $$p_{guess}(n_{i})\leq \frac{1}{2}.$$

Therefore, regardless of whether the adversary impersonates the prover in Case a (must output $\hat{n_{i}}$) or Case b (must disclose $n_{i}$), each verified position succeeds with probability at most 1/2, and mismatches occur with probability at least 1/2.

Let $W_{E}$ denote the mismatch count under impersonation. Then

(40) $$W_{E}\sim Binomial\left(D,\frac{1}{2}\right).$$

The FAR, i.e., the impersonation success probability under the threshold rule (29), is

(41) $$P_{FAR}\leq \mathrm{Pr}\left[W_{E}\leq \tau D\right]-\sum_{k=0}^{\left\lfloor \tau D\right\rfloor }\left(\begin{array}{c} D\\ k \end{array}\right){\left(\frac{1}{2}\right)}^{D}.$$

Using the standard Hamming-ball bound

(42) $$\sum_{k=0}^{\left\lfloor \tau D\right\rfloor }\left(\begin{array}{c} D\\ k \end{array}\right)\leq 2^{Dh_{2}(\tau )},$$

for 0<τ<1/2, where the binary entropy is

(43) $$h_{2}\left(\tau \right)=-\tau \log_{2}\tau -\left(1-\tau \right)\log_{2}\left(1-\tau \right).$$

Substitute (42) into (41):

(44) $$P_{FAR}\leq 2^{Dh_{2}\left(\tau \right)}\cdot 2^{-D}=2^{-D\left(1-h_{2}(\tau )\right)}.$$

Equation (42) bounds the number of transcripts within a Hamming-ball of radius τD, whose size scales as $2^{Dh_{2}\left(\tau \right)}$; this directly yields the exponent $1-h_{2}(\tau )$ in Equation (44).

If the adversary guesses the entire tested PSK segment correctly, impersonation succeeds trivially. Since each tested index uses two PSK bits $\left(b_{i},v_{i}\right)$, the tested segment has length 2D bits; thus

(45) $$P_{guess-PSK}=2^{-2D}.$$

A conservative total bound is

(46) $$P_{imp}\leq max\left\{2^{-2D},2^{-D\left(1-h_{2}\left(\tau \right)\right)}\right\}.$$

Equivalently, define the security strength κ (in bits) as

(47) $$\kappa =-\log_{2}P_{imp}\;\;\;\;\;⟹\;\;\;\;\;\kappa \geq \min\left\{2D,D\left(1-h_{2}(\tau )\right)\right\}.$$

To simultaneously achieve $P_{FRR}\leq \epsilon_{FRR}$ and $P_{imp}\leq 2^{-\kappa }$, one may:

① estimate $e_{auth}$ (or use $e_{1}^{U}$)
② choose $\tau >e_{auth}$ to ensure small $P_{FRR}$ via (32)~(36)
③ choose D so that $D\left(1-h_{2}(\tau )\right)\geq \kappa$ via (47)
④ ensure that the channel/decoy setting yields this D via (26).

The binary entropy $h_{2}\left(\tau \right)$ appears because the acceptance test effectively allows any transcript within a Hamming-ball of radius τD around the expected “honest” correlation pattern. The size of this Hamming-ball grows asymptotically as $2^{\left\{Dh_{2}\left(\tau \right)\right\}}$, which directly determines the exponent in the FAR bound. Equivalently, the factor $1-h_{2}\left(\tau \right)$ can be interpreted as a min-entropy rate of the verifier’s test against an adversary whose best per-trial matching probability is $\frac{1}{2}$; this yields a natural authentication security strength $\kappa =-\log_{2}P_{FAR}$. Similarly, the KL divergence term $KL\left(\tau ∥e_{auth}\right)$ in the Chernoff-type FRR bound quantifies statistical distinguishability between the honest-channel mismatch distribution (centered at $e_{auth}$) and the acceptance threshold τ. Operationally, it captures how rapidly the false-rejection probability decays with the number of verified trials D when $\tau >e_{auth}$, thereby making explicit the trade-off between robustness to channel noise and resistance to impersonation.

#### 3.3.4. PSK Management

The present protocol uses the PSK $A_{k}$ directly to determine the prepared states and Bob’s measurement bases in Section 2, while the nonce bits are revealed (Case b) or effectively exposed through prover messages (Case a). Therefore, long-term security requires explicit management of PSK reuse, particularly in practical implementations using WCP, where multi-photon emissions may allow additional information leakage over repeated sessions.

For one authentication execution that verifies $\left|I\right|=D$ positions, the amount of PSK material consumed is

(48) $$\left|A_{k}^{\left(I\right)}\right|=2D,$$

because each verified position uses two PSK bits $\left(b_{i},\;v_{i}\right)$. If mutual authentication (optional) is executed by running both verification directions (Case a and Case b) using disjoint PSK segments, the total PSK consumption becomes

(49) $$\left|A_{k,mutual}^{\left(I\right)}\right|=4D,$$

in the symmetric case where each direction uses D verified indices. (More generally, the total consumed bits equal $2D^{A\to B}+2D^{B\to A}$ when the verified-set sizes differ between directions.)

Verified-event rate and PSK budget. To avoid ambiguity, we explicitly distinguish the two authentication directions. We use the superscript U→V to denote the direction in which the prover is U and the verifier is V. In our protocol, B→A corresponds to Case a (Alice verifies Bob), and A→B corresponds to Case b (Bob verifies Alice). For a single authentication execution in direction U→V, let $I^{U\to V}$ be the verified index set used in the acceptance test of Section 3.3.1, and define $D^{U\to V}≜I^{U\to V}$ (number of verified events). Let $T_{exec}^{U\to V}$ denote the wall-clock duration over which these $D^{U\to V}$ verified events are accumulated (e.g., one “train” or one analysis window). We define the verified-event rate as

(50) $$R_{ver}^{U\to V}≜\frac{D^{U\to V}}{T_{exec}^{U\to V}}\;[events/s].$$

Since each verified index consumes two PSK bits $\left(b_{i},v_{i}\right)$, the PSK consumption rate for one-way authentication in direction U→V is

(51) $$R_{PSK}^{U\to V}=2R_{ver}^{U\to V}\;[bits/s].$$

If mutual authentication is executed by running both directions using disjoint PSK segments, the total PSK consumption rate becomes

(52) $$R_{PSK}^{\left(mutual\right)}=2\left(R_{ver}^{A\to B}+R_{ver}^{B\to A}\right)\approx 4R_{ver}\;[bits/s].$$

In the WCP setting, for a pulse with mean photon number $x\in \left\{\mu ,\;\nu \right\}$, the probability of emitting at least two photons is

(53) $$P_{\geq 2}\left(x\right)=1-\mathrm{Pr}\left[0\right]-\mathrm{Pr}\left[1\right]=1-e^{-x}\left(1+x\right).$$

A conservative operational stance is that any authentication indices associated with multi-photon emissions can become progressively less secure under repeated use (e.g., an adversary may keep an extra photon and correlate later public information related to nonce disclosure/announcement). This motivates a strict or bounded PSK reuse policy.

Information-theoretic PSK refresh using QKD keys. A robust information-theoretic refresh policy is available when authentication is executed during BB84-type QKD operation. Let $K_{QKD}^{\left(s\right)}$ denote the secret key distilled from QKD session s (after error correction and privacy amplification). Define the PSK material for the next authentication epoch $\left(s+1\right)$ by extracting fresh bits from $K_{QKD}^{\left(s\right)}$:

(54) $$A_{k}^{\left(s+1\right)}\leftarrow first\;2D^{\left(s+1\right)}\;bits\;of\;K_{QKD}^{\left(s\right)},$$

where $D_{tot}^{\left(s+1\right)}=D^{\left(s+1,\;U\to V\right)}$ for one-way operation in direction U→V, and $D_{tot}^{\left(s+1\right)}=D^{\left(s+1,\;A\to B\right)}+D^{\left(s+1,\;B\to A\right)}$ for mutual operation. In the mutual case, the extracted $2D_{tot}^{\left(s+1\right)}$ bits are split into two disjoint PSK segments (domain separation) assigned to the two directions to avoid cross-direction reuse. This requires the key-budget condition

(55) $$\left|K_{QKD}^{\left(s\right)}\right|\geq 2D^{\left(s+1\right)}+l_{MAC}^{\left(s+1\right)},$$

where $l_{MAC}^{\left(s+1\right)}$ accounts for any additional secret bits reserved to authenticate classical messages, if applicable. In rate form, letting $R_{QKD}$ denote the net secret-key generation rate available for PSK refresh and $R_{MAC}$ the classical-authentication key consumption rate, a sustainable policy requires $R_{QKD}\geq R_{PSK}+R_{MAC}$, with $R_{PSK}=2R_{ver}^{U\to V}$ (one-way) or $R_{PSK}=2\left(R_{ver}^{A\to B}+R_{ver}^{B\to A}\right)$ (mutual).

Under standard QKD security, this yields fresh PSK segments independent of prior public transcripts, enabling “one-time consumption” of PSK symbols across sessions. If long PSK storage is operationally undesirable, a computationally motivated alternative is to maintain a master secret $K_{master}$ of λ bits and derive a per-session PSK using a keyed derivation function. Specifically, let KDF(·) denote a key derivation function (or equivalently a pseudorandom function (PRF) instantiated KDF) that, for a fixed secret key, maps a public “context” string to an output bit string that is computationally indistinguishable from uniform to any efficient adversary without the key. In our setting, the context binds the derived PSK to the session and the authentication direction to ensure domain separation. The per-session PSK is then derived as

(56) $$A_{k}^{\left(s\right)}=KDF\left(K_{master},sid=s∥role\right),$$

where ‘role’ distinguishes protocol directions (e.g., Alice → Bob vs. Bob → Alice) to avoid cross-protocol/key reuse. Here, ‘sid=s’ denotes a public session identifier (e.g., a monotonically increasing counter or a timestamp) that is unique per authentication execution, and ‘role’ is a public label that specifies the authentication direction (e.g., “A → B” or “B → A”) for domain separation. The derived string $A_{k}^{\left(s\right)}$ is then parsed into D two-bit symbols to define the per-trial basis/value bits used in the protocol:

(57) $${\left(A_{k}^{\left(s\right)}\right)}_{i}=\left(b_{i}^{\left(s\right)},v_{i}^{\left(s\right)}\right),\;i=1,\ldots ,D,$$

with $\left(b_{i}^{\left(s\right)},v_{i}^{\left(s\right)}\right)$ assigned from consecutive bit pairs of $A_{k}^{\left(s\right)}$ (hence $\left|A_{k}^{\left(s\right)}\right|=2D$ bits for one-way authentication, or 4D bits for mutual authentication).

The master secret is then refreshed using newly generated QKD keys:

(58) $$K_{master}\leftarrow KDF\left(K_{master},K_{QKD}^{\left(s\right)}\right).$$

One may alternatively derive per-session PSK material from a master secret via a KDF/PRF; however, this would introduce computational assumptions and is therefore not part of the information-theoretic end-to-end security chain emphasized in this work.

In summary, the protocol binds the PSK $A_{k}$ directly to state preparation and basis choice. Since WCP implementations introduce a nonzero multi-photon probability $P_{\geq 2}(x)$, long-term soundness is best supported by treating PSK symbols as consumable resources (size 2D per direction) and enforcing explicit refresh rules, preferably via information-theoretic extraction from QKD-generated keys.

#### 3.3.5. Complexity–Performance Trade-Off Metrics

To make the implementation trade-off explicit, we summarize the relationship between authentication performance and operational overhead using a small set of system-level metrics. We quantify authentication security strength by

(59) $$\kappa ≜-\log_{2}\left(P_{FAR}\right)\;[bits],$$

where $P_{FAR}$ is the false-acceptance (impersonation) probability under the acceptance rule in Section 3.3.1. Using the Hamming-ball bound applied in Section 3.3, we have

(60) $$P_{FAR}\leq 2^{-D\left(1-h_{2}\left(\tau \right)\right)}⟹\kappa \geq D\left(1-h_{2}\left(\tau \right)\right),$$

where $D=\left|I\right|$ is the number of verified indices and $h_{2}\left(\cdot \right)$ is the binary entropy function. We quantify robustness/usability by the false-rejection probability $P_{FRR}$, which for an honest prover with per-trial mismatch probability $e_{auth}$ satisfies the Chernoff-type bound

(61) $$P_{FRR}=\mathrm{Pr}\left[W>\tau D\right]\leq \exp\left(-D\;KL\left(\tau ∥e_{auth}\right)\right),$$

where $KL\left(a∥b\right)=a\ln\frac{a}{b}+\left(1-a\right)\ln\frac{1-a}{1-b}$.

**Figure 2:**
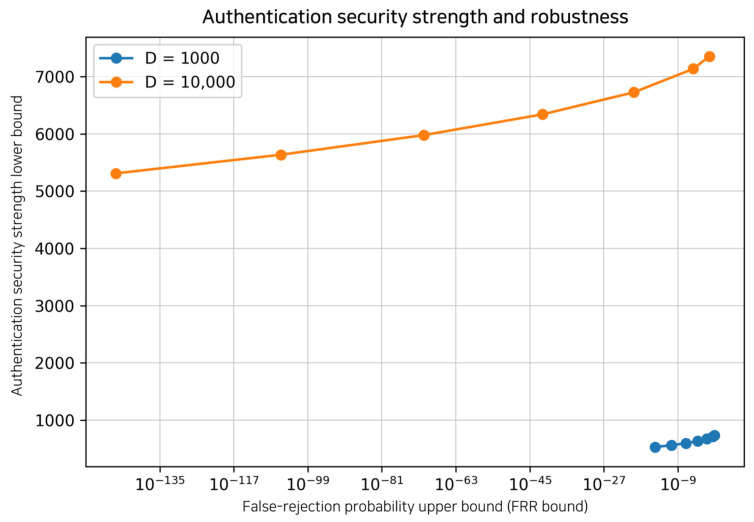
Trade-off between authentication security strength and robustness controlled by the threshold τ.

Operationally, we define throughput as the verified-event rate $R_{ver}=D/T_{exec}$ (events/s). For one-way authentication, the corresponding PSK consumption rate is $R_{PSK}=2R_{ver}$ (bits/s) because each verified index consumes two PSK bits. Mutual authentication is optional; when both directions are executed using disjoint PSK segments, the total consumption becomes $R_{PSK}^{mutual}=2\left(R_{ver}^{A\to B}+R_{ver}^{B\to A}\right)\approx 4R_{ver}$. Table 1 summarizes these complexity and overhead terms. Figure 2 visualizes the fundamental trade-off controlled by the threshold τ: decreasing τ strengthens security (larger κ) but increases false rejections (larger $P_{FRR}$), while choosing τ moderately above the observed honest mismatch level $e_{auth}$ yields low FRR with a quantifiable security exponent.

The names and definitions of the variables used in this study are summarized in Table 2 for clarity.

## 4. Realization Results on Communication Channels

In this section, we describe the experimental implementation of the proposed QEA protocol over a deployed optical network. Although the protocol is theoretically defined based on single-photon sources, the implementation utilizes WCPs and the decoy-state method to ensure security against photon-number-splitting attacks, as discussed in the security analysis. The quantum states employed in this experiment are four polarization states of photons.

### 4.1. Experimental Configuration

**Figure 3:**
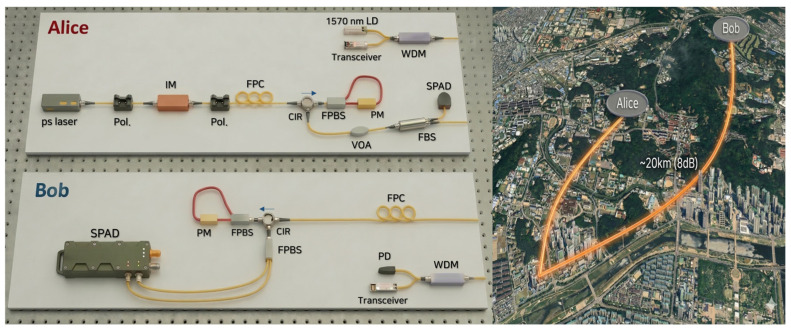
Experimental setup for proposed QEA implementation. At Alice (transmitter), picosecond optical pulses are generated and shaped using an intensity modulator, prepared with well-defined polarization, and encoded using an all-fiber module incorporating a fiber polarizing beam splitter (FPBS) and a phase modulator. A classical synchronization channel from a 15xx-nm laser diode (LD; clock) is wavelength-multiplexed using wavelength-division multiplexing (WDM). A fiber beam splitter (FBS) provides an optional monitoring tap directed to single-photon avalanche diodes (SPAD). The signals are transmitted through a field-deployed fiber span of approximately 20 km with an overall loss of about 8 dB. At Bob (receiver), WDM separates the classical channels; the clock channel is detected with a photodiode (PD) and interfaced through a classical transceiver for synchronization. Separately, the quantum channel is polarization-aligned using a fiber polarization controller (FPC), analyzed in an FPBS-based polarization decoding system, and detected with SPADs.

In this implementation, Alice and Bob utilized photon polarization states ($\left|\left.D\right\rangle \right.$, $\left|\left.A\right\rangle \right.$, $\left|\left.R\right\rangle \right.$, $\left|\left.L\right\rangle \right.$). These polarization states correspond to the protocol’s quantum states $\left|\left.0\right\rangle \right.$, $\left|\left.1\right\rangle \right.$, $\left|\left.+\right\rangle \right.$, $\left|\left.-\right\rangle \right.$, respectively. We assumed that the PSK was securely distributed between Alice and Bob beforehand. Figure 3 illustrates the experimental setup for the quantum authentication system, which consists of Alice, Bob, a quantum channel, and a classical channel.

#### 4.1.1. Transmitter (Alice)

Alice encodes the quantum states according to the PSK and transmits them to Bob. The decoy states are randomly selected by Alice. Alice’s setup comprises a light source, a quantum state encoding device, a mean photon number monitor, clock synchronization, and classical communication modules.

- Light Source: We used a picosecond pulsed laser (ps laser) operating at a wavelength of 1550 nm with a spectral bandwidth of <1 nm and a temporal pulse width of approximately 500 ps. The laser generates pulses for signal and decoy states, while no pulse is generated for the vacuum state.
- Intensity Modulation: An intensity modulator (IM) determines the signal and decoy intensities. The IM is stabilized at the minimum transmission point using a bias controller and a 1570 nm pilot laser. When generating signal states, the IM is inactive (allowing the pulse to pass); for decoy states, voltage is applied to attenuate the pulse intensity. An optical attenuator is then used to reduce the pulses to the single-photon level before they enter the quantum channel.
- State Encoding: The quantum state encoding device consists of a Sagnac interferometer and a phase modulator (PM) [35]. The incident photon polarization is $\left|\left.D\right\rangle \right.$, which is converted into one of the four states ($\left|\left.D\right\rangle \right.$, $\left|\left.A\right\rangle \right.$, $\left|\left.R\right\rangle \right.$, $\left|\left.L\right\rangle \right.$) depending on the voltage applied to the PM. During encoding, Alice applies the random bit-flip operation required by the authentication protocol.
- Monitoring: A mean photon number monitor, consisting of a 50:50 beam splitter and a single-photon detector (SPD), measures the presence of photons in the pulses sent to Bob. Assuming a Poissonian photon number distribution, this setup estimates the mean photon number for signal and decoy states.

#### 4.1.2. Receiver (Bob)

Bob is responsible for decoding and measuring the incoming quantum states. His setup includes a fiber polarization controller (FPC), a polarization state decoding device, a polarization measurement unit, a trigger detector, and a clock synchronization module.

- Polarization Control: The FPC compensates for polarization drifts caused by the quantum channel, converting the arbitrary unitary transformation induced by the fiber into an identity operation.
- Decoding and Measurement: Bob’s decoding device is structurally identical to Alice’s encoder. Based on the basis information derived from the locally stored PSK, Bob applies a voltage to his PM to switch between the z-basis (|D⟩, |A⟩) and the x-basis (|R⟩, |L⟩). The photons are then measured using a polarization beam splitter (PBS) and single-photon avalanche diodes (SPADs) with a detection efficiency of approximately 20%.
- Synchronization: Both entities are synchronized to a 10 MHz clock. Alice transmits a trigger signal alongside the quantum signal. Bob detects this trigger using a photodiode (PD) and a pre-amplifier to maintain synchronization.

#### 4.1.3. Communication Channels

The system utilizes both quantum and classical channels, similar to a QKD architecture.

- Classical Channel: Assumed to be an authenticated public channel where an eavesdropper can read but not modify messages. It is used for clock signals, trigger signals (via ns pulse laser and PD), and classical information exchange (via TCP/IP).
- Quantum Channel: The experiment utilized the deployed optical network connecting Alice and Bob via a detour route. The physical distance is approximately 20 km. While the theoretical loss for this distance is around 4 dB, the measured optical loss was approximately 8 dB.

### 4.2. Experimental Conditions

Prior to the execution of the protocol, Alice and Bob are assumed to share a PSK of size 1,048,576 bits (524,288 bits ×2). One “train” sent by Alice consists of 524,288 qubits. The mean photon numbers for signal and decoy states were set to approximately 0.5 and 0.15, respectively. The probabilities for sending signal, decoy, and vacuum states were configured to approximately 0.88, 0.10, and 0.02. Table 3 summarizes the experimental parameters.

### 4.3. Experimental Results and Analysis

The proposed quantum authentication protocol was implemented on a physically deployed network.

#### 4.3.1. Key Rate and QBER Performance

**Figure 4:**
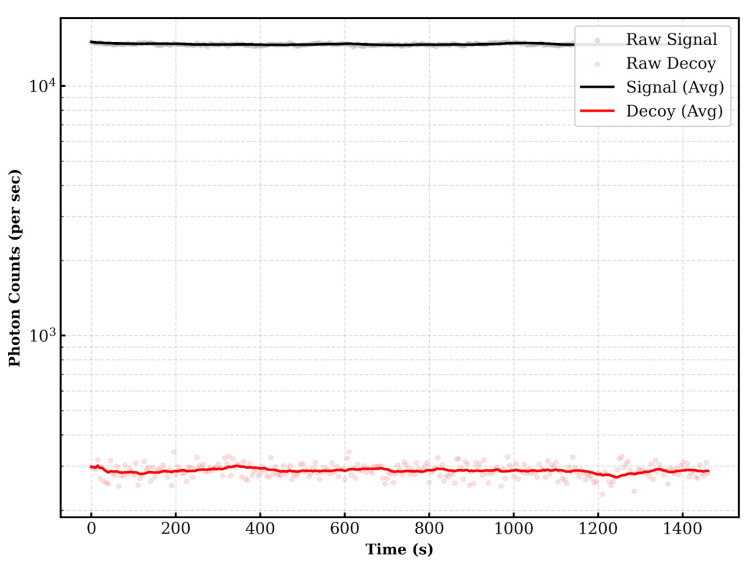
Sifted key rate over time.

In a practical authentication scenario, reusing the same PSK is insecure. However, for the purpose of validating the implementation on a deployed fiber, we transmitted one train (524,288 qubits) every 5 s using the pre-distributed keys. Figure 4 illustrates the sifted key rate over a duration of approximately 1500 s. Since Alice and Bob share the PSK, they always measure in the same basis. Consequently, the volume of the sifted key is identical to that of the raw key. The results show a stable key rate over time. The difference in key generation rates between signal and decoy states is due to their different mean photon numbers.

**Figure 5:**
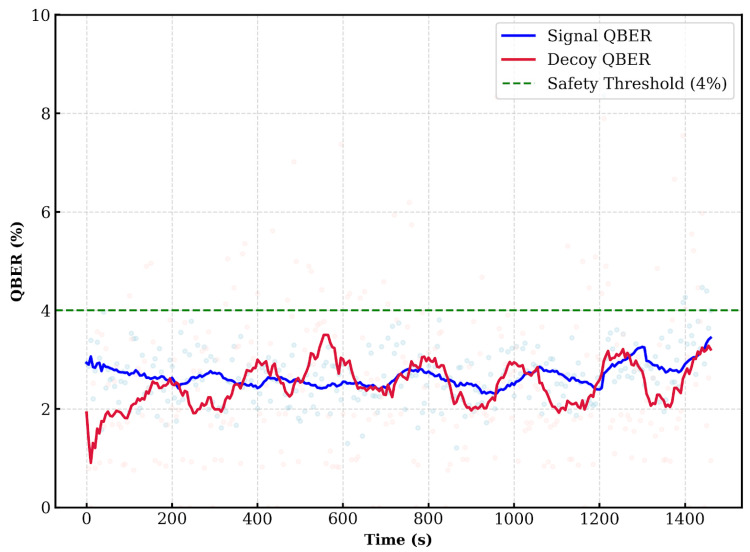
QBER when all bit-flip information is disclosed. The green dashed line indicates the operational safety threshold (δ≈4%). This value is based on the acceptance rule defined in Section 3.3.1, which requires the QBER to remain below a specific limit to minimize the FAR. As validated in the experimental results, the system maintains a QBER between 1% and 4% under normal conditions, confirming that a 4% threshold provides a robust margin to distinguish legitimate signals from potential attacks.

Figure 5 presents the QBER for signal and decoy states. The QBER was calculated using the PSK and Alice’s bit-flip information. Due to the lower mean photon number and probability of decoy states, the sifted key volume for decoys is smaller, resulting in larger statistical fluctuations in the QBER compared to the signal states.

#### 4.3.2. Case A: Alice as Verifier, Bob as Prover

We first evaluated the scenario where Alice acts as the verifier and Bob as the prover. Alice measured the key rate and QBER using her local PSK, her bit-flip records, and the measurement results announced by Bob.

**Figure 6:**
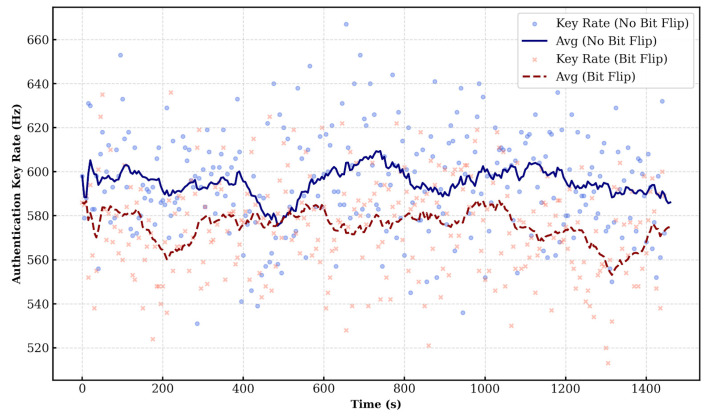
Key rate (Verifier: Alice, Prover: Bob).

Figure 6 shows the key rate for this configuration. Since Alice performs the bit-flip operation with a 50% probability, the key generation rates for the “no bit flip” and “bit flip” cases are similar and remain stable over time.

**Figure 7:**
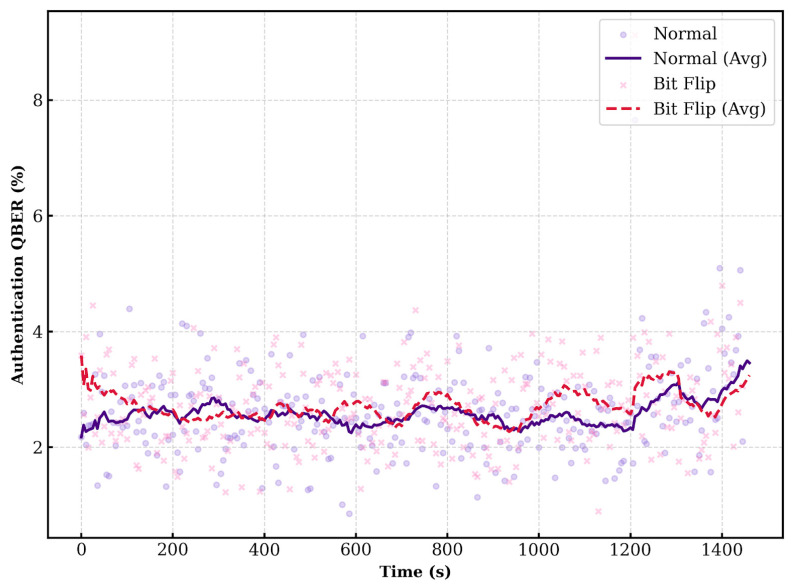
QBER (Verifier: Alice, Prover: Bob).

Figure 7 displays the QBER measured by Alice. The QBER mostly remains within the range of 1% to 4%. Instances where the QBER deviates from this range tend to occur simultaneously for both “no bit flip” and “bit flip” cases. Alice accepts Bob as authenticated if the observed QBER falls within the pre-defined security threshold.

#### 4.3.3. Case B: Bob as Verifier, Alice as Prover

**Figure 8:**
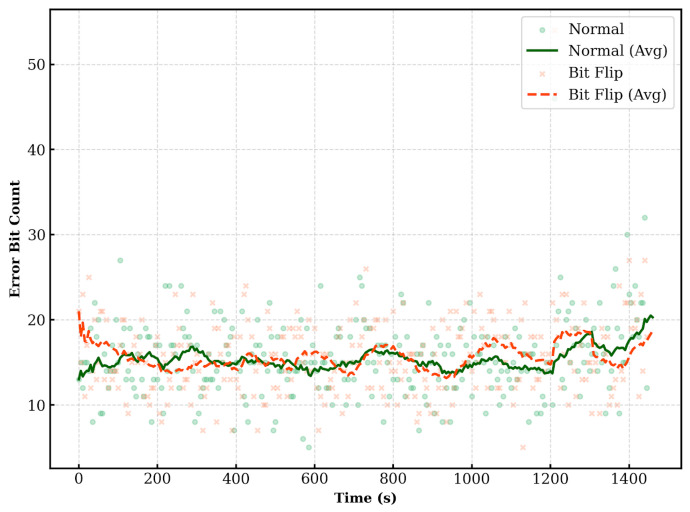
Key rate (Verifier: Bob, Prover: Alice).

Next, we evaluated the reverse scenario where Bob acts as the verifier and Alice as the prover. In this case, Alice discloses her bit-flip information to Bob. Bob then calculates the key rate and QBER based on Alice’s disclosed information, his measurement outcomes, and the PSK. Figure 8 shows the key rate results measured by Bob. Similar to the previous case, the key rates for “no bit flip” and “bit flip” are comparable due to the 50% flip probability.

**Figure 9:**
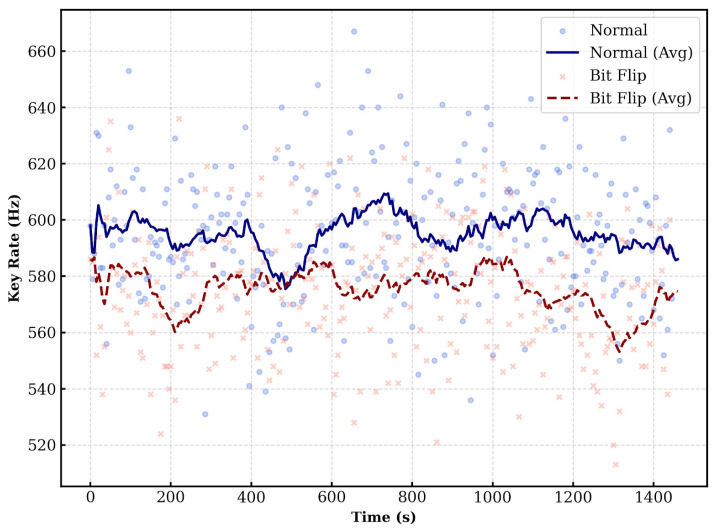
QBER (Verifier: Bob, Prover: Alice).

Figure 9 presents the QBER measured by Bob using the bit-flip information provided by Alice. Consistent with the Alice-verifier scenario, the QBER is generally observed between 1% and 4%. Bob authenticates Alice based on this QBER value.

The experimental results confirm that the proposed quantum authentication protocol can be stably implemented over a real-world optical network. While the key rate remained constant, the QBER showed fluctuations between 1% and 4%.

## 5. Conclusions

In this work, we implemented and field-tested a PSK-seeded quantum entity authentication protocol designed to operate within BB84-type QKD terminal workflows without requiring modifications to the optical hardware. Using weak coherent pulses with a standard decoy-state setting (signal/decoy mean photon numbers of approximately 0.5/0.15 and sending probabilities of 0.88/0.10/0.02), we demonstrated stable operation over a deployed fiber link of about 20 km with an overall loss of ~8 dB. Across both verification directions, the measured QBER remained typically within the 1~4% range under normal conditions, supporting a practical acceptance threshold consistent with the decision rule analyzed in Section 3.3.1. A significant advantage of the proposed protocol is not only its practical feasibility within existing quantum network infrastructures, but also its support for runtime authentication-key updating within the QKD operating workflow, thereby reducing long-term reliance on a single static authentication key.

The present security claims are conditioned on an authenticated (but not encrypted) classical channel and on the assumed secrecy/refresh of the PSK; moreover, the analysis primarily addresses channel-level impersonation (e.g., intercept–resend-type disturbance) and conservative WCP leakage handling via decoy-state bounds, rather than providing a fully composable finite-key proof under general non-i.i.d. temporal correlations. In addition, device-level side-channel attacks (e.g., detector blinding, Trojan-horse probing of modulators, and calibration attacks) are not treated here and require countermeasures that are complementary to the protocol logic. Future work will (i) map the same authentication logic to phase-encoding implementations commonly used in standard QKD terminals, (ii) strengthen the finite-size treatment using tighter finite-key methods that can accommodate drift and correlated trials, and (iii) integrate and experimentally validate practical device-countermeasure techniques to extend robustness under realistic adversarial models.

A significant advantage of the proposed protocol is its practical feasibility within existing quantum network infrastructures. We demonstrated that the protocol can be deployed on standard QKD systems without requiring any modifications to the optical hardware. By utilizing polarization states for encoding and employing the decoy-state method to counter PNS attacks, the system effectively utilizes WCP while maintaining security guarantees comparable to those of single-photon sources. Although this study exclusively demonstrates and analyzes polarization encoding, the protocol is equally applicable to phase-based encoding—analogous to standard QKD implementations—with comparable performance expectations. This compatibility ensures that physical-layer authentication can be seamlessly integrated as an intrinsic feature of future quantum communication terminals.

The theoretical analysis and experimental results confirm the protocol’s capability to provide robust mutual authentication. Our security analysis established that an adversary performing an intercept–resend attack introduces a detectable error rate due to the lack of the PSK, thereby allowing the verifier to reject illegitimate entities with a probability that converges to unity. Furthermore, we validated the protocol over a deployed optical fiber network spanning approximately 20 km. The experimental results showed stable key rates and QBER within the required security thresholds, proving the protocol’s resilience against environmental decoherence and channel attenuation. These findings underscore the feasibility of integrating QEA into next-generation networks, thereby ensuring a holistic security architecture for the global quantum internet.

## Figures and Tables

**Table 1:** Complexity–performance summary and operational overhead metrics.

Category	Metric/Item	One-Way Auth.	Mutual Auth.
Security strength	$\kappa ≜-log_{2}\left(P_{FAR}\right)\left[bits\right]$	$\kappa \geq D(1-h_{2}\left(\tau \right))$	Per direction $\kappa^{A\to B}$ and $\kappa^{B\to A}$
Robustness	$P_{FRR}=Pr\left[W>\tau D\right]$	$P_{FRR}\leq exp\left(-D\;KL(\tau ∥e_{auth})\right)$	Same bound per direction with its own $D^{A\to B},e_{auth}^{A\to B}$ and $D^{B\to A},e_{auth}^{B\to A}$
Throughput	Verified-event rate $(R_{ver}^{U\to V}$)	$R_{ver}^{U\to V}=D^{U-V}/T_{exec}^{U\to V}$ [events/s]	$R_{ver}^{A\to B}$ and $R_{ver}^{B\to A}$ averaged (symmetric)
Secret-key overhead	PSK consumption	$R_{PSK}^{U\to V}=2R_{ver}^{U\to V}$ [bits/s]	$R_{PSK}^{mutual}\approx 4R_{ver}$
Classical-auth. overhead	Key budget term	Include $R_{MAC}$ [bits/s] if information-theoretic MAC is used on classical messages	Same, potentially doubled if both directions incur separate authenticated transcripts
Computation	Per verified event	Lightweight O(1) bit operations (XOR/bit compare) plus optional MAC verification; total O(D) per execution	Same per direction; total $O(D^{A\to B}+D^{B\to A})$
Communication	Classical transcript	Nonce announcements/requests and acceptance decision; overhead scales linearly with execution count	Two direction-specific transcripts (domain separated), same message types repeated
Hardware	Added optical components	None (reuses BB84-type terminal optics and basis control)	

**Table 2:** Summary of key variables for the PSK-seeded QEA protocol, WCP/decoy-state model, and finite-size authentication bounds. Unless explicitly stated otherwise, all scalar system parameters and rates used in bounds and plots (e.g., $Q_{x},e_{x},D,\tau ,\kappa ,R_{ver},R_{PSK}$) are real-valued. Complex quantities appear only in quantum-state vectors and operators (Dirac notation), not in the operational performance parameters.

Symbol	Name	Description
$A_{k}$	Pre-shared authentication key	PSK used for entity authentication
$S\left(\cdot \right)$	BB84 state mapping function	Mapping from $\left(b_{i},v_{i}\;\right)$
I	Verified index set	Set of indices used in the acceptance test (verified events)
$\Delta_{i}$	Mismatch indicator	Indicator variable for mismatch at index i
$e_{auth}$	Honest mismatch probability	Per-trial mismatch probability under honest operation (noise level)
$P_{FRR}$	False rejection probability	Probability that an honest prover is rejected
$P_{FAR}$	False acceptance probability	Probability that an adversary is accepted (impersonation success)
$KL\left(\tau ∥e_{auth}\right)$	Binary KL divergence	Binary relative entropy used as a Chernoff exponent in FRR bounds
κ	Authentication security strength	Security strength in bits, typically $\kappa ≜-\log_{2}\left(P_{FAR}\right)$
s	QKD session index	Index of QKD session used for PSK refresh budgeting
$K_{QKD}^{\left(s\right)}$	Distilled QKD key	Final secret key distilled from QKD session s (after EC and PA)
$T_{exec}^{U\to V}$	Execution time window	Time window duration used to accumulate verified events in direction U→V
$R_{ver}^{U\to V}$	Verified-event rate	Verified-event rate in direction U→V [events/s]
$R_{PSK}$	PSK consumption rate	PSK consumption rate [bits/s]
$R_{QKD}$	QKD key rate	Net QKD secret-key generation rate available for PSK refresh [bits/s]
$R_{MAC}$	MAC key consumption rate	Secret-key consumption rate for authenticating classical messages (e.g., IT-MAC)

**Table 3:** Experimental conditions for the implementation of the quantum authentication protocol.

Parameter		Value
Average photon number	Signal	~0.5
	Decoy	~0.15
Intensity probabilities	Signal	0.88
	Decoy	0.10
	Vacuum	0.02
PSK size		1,048,576 bits
One train length		524,288 qubits
Alice’s bit flip probability		50%
Quantum channel length		~20 km
Quantum channel loss		~8 dB

## Data Availability

The original contributions presented in this study are included in the article. Further inquiries can be directed to the corresponding author.
